# The Applicability of Novel Predictor of Intracranial Hemorrhage in Patients With Atrial Fibrillation in the Contemporary Real‐World Clinical Practice

**DOI:** 10.1002/clc.70078

**Published:** 2025-01-13

**Authors:** Naoya Kataoka, Teruhiko Imamura

**Affiliations:** ^1^ Second Department of Internal Medicine University of Toyama Toyama Japan

**Keywords:** anticoagulation therapy, bleeding, HAS‐BLED score, heart failure

To the Editor,

Major bleeding, including intracranial hemorrhage (ICH), is a significant complication in patients with non‐valvular atrial fibrillation (NVAF) undergoing treatment with oral anticoagulants (OACs). The authors proposed a novel risk score for predicting ICH in NVAF patients, incorporating variables such as age, sex, nonsmoking status, renal replacement therapy, and OAC use [1]. However, several critical concerns merit discussion.

The authors compared their proposed risk score with the established HAS‐BLED score [1], a widely utilized tool for predicting not only ICH but also other major bleeding events classified as ≥ BARC 3b [2]. Unlike the novel score, the HAS‐BLED score includes parameters such as hepatic dysfunction and the use of antiplatelet agents. Consequently, employing the HAS‐BLED score as a comparator may not fully capture the nuances of the novel score's predictive capability for ICH specifically.

Patients with NVAF are susceptible to a range of complications, including thromboembolic events and heart failure. A noteworthy concern is the potential applicability of the novel risk score in predicting these broader complications. Furthermore, in the authors' study, only 6.7% of the cohort were treated with direct oral anticoagulants (DOACs) [1], which currently represent the predominant class of anticoagulants in clinical practice [3]. This limited representation raises questions about the generalizability of the score to patients receiving DOACs, warranting further validation.

Additionally, prior literature advises against the use of OACs in patients undergoing renal replacement therapy due to heightened bleeding risks [4]. Excluding such patients from the construction of risk scores may be more appropriate to ensure clinical relevance and applicability.

## Ethics Statement

The authors have nothing to report.

## Consent

The authors have nothing to report.

## Conflicts of Interest

The authors declare no conflicts of interest.

## Data Availability

The authors have nothing to report.
